# Finite Element Analysis of Implant Stability Quotient (ISQ) and Bone Stresses for Implant Inclinations of 0°, 15°, and 20°

**DOI:** 10.3390/ma18071625

**Published:** 2025-04-02

**Authors:** Mario Ceddia, Tea Romasco, Giulia Marchioli, Luca Comuzzi, Alessandro Cipollina, Adriano Piattelli, Luciano Lamberti, Natalia Di Pietro, Bartolomeo Trentadue

**Affiliations:** 1 Department of Mechanics, Mathematics and Management, Polytechnic University of Bari, 70125 Bari, Italy; marioceddia1998@gmail.com (M.C.); luciano.lamberti@poliba.it (L.L.); bartolomeo.trentadue@poliba.it (B.T.); 2 Department of Medical, Oral and Biotechnological Sciences, “G. d’Annunzio” University of Chieti-Pescara, 66100 Chieti, Italy; tea.romasco@unich.it (T.R.); giuliamarchioli912@gmail.com (G.M.); 3 Center for Advanced Studies and Technologies (CAST), “G. d’Annunzio” University of Chieti-Pescara, 66100 Chieti, Italy; 4 Independent Researcher, 31020 San Vendemiano, Italy; luca.comuzzi@gmail.com; 5 Independent Researcher, 92019 Sciacca, Italy; alexandros1960@libero.it; 6 School of Dentistry, Saint Camillus International University of Health and Medical Sciences, 00131 Rome, Italy; apiattelli51@gmail.com; 7 Facultad de Medicina, UCAM Universidad Católica San Antonio de Murcia, 30107 Murcia, Spain

**Keywords:** dental implants, finite element analysis (FEA), implant stability quotient (ISQ), inclination, polyurethane, stress distribution

## Abstract

This study aimed to utilize finite element analysis (FEA) to evaluate the primary stability of Cyroth dental implants (AoN Implants Srl, Grisignano di Zocco, Italy) under various biomechanical conditions, including different implant inclinations (0°, 15°, and 20°) and bone densities (D3 and D4). By comparing these results with those obtained from in vitro tests on polyurethane blocks, the study sought to determine whether FEA could provide stability information more quickly and efficiently than in vitro methods. The research involved correlating dental implant micro-mobility with the implant stability quotient (ISQ) using FEA to simulate the mechanical behavior of implants and the surrounding bone tissue. Additionally, the study assessed the error in ISQ value detection by comparing FEA results with in vitro tests on polyurethane blocks conducted under the same experimental conditions. Both the FEA simulations and in vitro experiments demonstrated similar trends in ISQ values. For the D3 bone block simulated by FEA, the difference from the in vitro test was only 1.27%, while for the D2 bone, the difference was 2.86%. The findings also indicated that ISQ increases with implant inclination and that bone quality significantly affects primary stability, with ISQ decreasing as bone density diminishes. Overall, this study showed that ISQ evaluation for dental implants can be effectively performed through FEA, particularly by examining micro-movements. The results indicated that FEA and in vitro polyurethane testing yielded comparable outcomes, with FEA providing a faster and more cost-effective means of assessing ISQ across various clinical scenarios compared to in vitro testing.

## 1. Introduction

The primary stability of dental implants is crucial for their long-term success, as it directly affects osseointegration—the process by which the surrounding bone firmly bonds to the implant [1,2]. According to Misch [3], a “stable implant” is defined by the microscopic interactions between the implant surface and the bone, along with minimal movement when subjected to forces ranging from 1 to 500 g in both horizontal and vertical directions. Various factors contribute to implant stability and can be classified into biological and biomechanical elements. Biological factors include variables such as bone density, the volume of residual bone after extraction, and parafunctional habits, all of which influence bone-to-implant contact (BIC). In contrast, biomechanical factors include aspects such as implant design, chemical composition, materials used, surface treatments, and loading durations. These elements play a vital role in the distribution of forces on the implant and its ability to withstand loads and stresses, thereby improving implant stability [4,5].

Primary stability is a crucial determinant of an implant’s success when subjected to immediate loading [6,7]. Moreover, secondary stability, which develops in the weeks following implant placement, is equally critical for ensuring sustained osseointegration and preventing implant movement that might hinder the healing process [8]. In clinical practice, resonance frequency analysis (RFA) is employed to assess primary stability, specifically by quantifying the implant stability quotient (ISQ) [9,10,11,12]. This metric, ranging from 0 to 100, indicates that higher values correspond to greater stability, thereby providing valuable insights into the implant’s stability. Such insights are essential for evaluating the patient’s healing process and the overall success of the treatment [12]. RFA can be conducted in vivo to reflect real-time primary stability during the post-implantation insertion and healing phases [13,14] or through in vitro testing with polyurethane models that mimic various bone densities [15,16]. For instance, a recent study using polyurethane blocks evaluated the insertion torque (IT), removal torque (RT), and ISQ values of implants of varying lengths [17]. This research found a significant correlation between implant length and primary stability, showing that longer implants exhibited higher IT, RT, and ISQ values. Another study by Comuzzi et al. [18] compared the primary stability of different implant designs—cylindrical and conical—within low-density polyurethane foam blocks (10 and 20 pounds per cubic foot, PCF). The results indicated that higher IT and RT values were linked to the higher-density block, suggesting enhanced implant stability in the densest substrate, corroborating findings from existing literature [19,20]. Furthermore, conical implants showed higher ISQ values than cylindrical implants, emphasizing the substantial impact of both bone density and implant geometry on primary stability.

It is also important to note that the biomechanical properties of the surrounding bone tissue, including mineral density and bone microstructure, significantly affect implant stability [21]. A study by Pammer et al. [22] found a decrease in ISQ values that corresponded with bone density, suggesting that denser bone improves implant stability. Conversely, areas with lower bone density may face increased stress concentrations, which can raise the risk of local fractures or implant failure [23].

Currently, there are various methodologies for evaluating bone stresses induced by implant placement [24,25,26,27]. Among these techniques, photoelastic analysis using photoelastic materials allows for the visualization and measurement of stress within specimens that simulate bone tissue [23]. By applying a load to the sample, the photoelastic method generates light interference patterns that correspond to different stress intensities. However, the applicability of this technique is limited, as it is only effective with materials that exhibit photoelastic properties. Many materials used in dentistry or engineering do not have these properties, restricting the technique’s utility. Additionally, while photoelasticity provides qualitative insights into stress, converting this information into precise quantitative values can be complex and may require additional modeling.

Recently, the finite element method (FEM) has become a popular approach in dental implantology, allowing for the modeling of intricate and complex geometries that may be challenging to analyze using photoelasticity [23]. FEM enables the selection of various materials with distinct mechanical properties and the application of advanced constitutive models, including anisotropic, plastic, or viscoelastic materials [28,29,30]. Current studies have utilized FEM to evaluate bone stresses under different loading scenarios and implant configurations. For example, research by Pirmoradian et al. [31] revealed that the maximum stress distribution in dental implants mainly occurs in the contact area between the implant and the abutment. Average stresses were observed in cortical bone, while lower values were noted in trabecular bone, consistent with its reduced elastic stiffness. Another study by Kong et al. [32] demonstrated that increasing the implant diameter significantly reduces the maximum von Mises stress in cortical bone tissue, achieving a 65.8% reduction under buccolingual loading. Similarly, in cancellous bone, the maximum stress experienced under axial loading decreased by 71.5% with increasing implant length. Ceddia et al. [33] showed that the oblique placement of an implant in less dense bone with an inadequate amount of cortical bone resulted in increased peri-implant stress. However, there is a significant lack of literature addressing the effect of implant inclination on primary stability as measured by the ISQ index. The vertical and transverse forces generated during mastication produce axial forces and bending moments, leading to stress gradients and deformations in both the implant and surrounding bone [34,35]. Moreover, finite element studies suggest that stresses on peri-implant tissues are intensified when an implant is tilted during insertion [36,37,38].

The objective of this study was to develop a method using finite element analysis (FEA) simulations to establish a direct correlation between FEA outcomes and ISQ measurements for tilted implants (angles of 0°, 15°, and 20°) inserted into bone blocks of varying densities. This method is designed to be user-friendly for both laboratory researchers and clinical practitioners routinely involved in ISQ assessment.

## 2. Materials and Methods

### 2.1. Modeling

**Figure 1:**
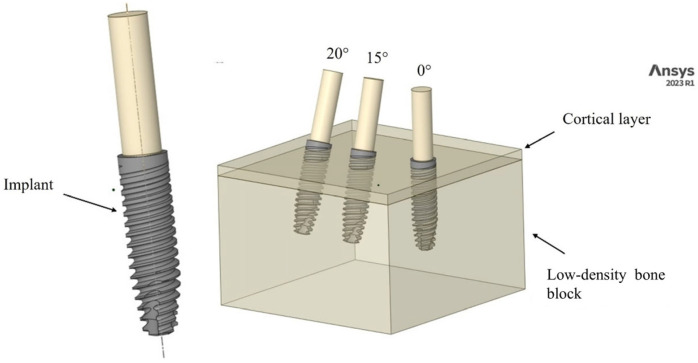
Modeling and positioning of Cyroth implants (AoN Implants Srl, Grisignano di Zocco, Italy) within the bone block at angles of 0°, 15°, and 20°.

The FEM approach was employed to evaluate and compare the ISQ results from Cyroth implants (4 mm × 15 mm, AoN Implants Srl, Grisignano di Zocco, Italy). A three-dimensional (3D) model was constructed using computer-aided design (CAD) software (Autodesk Inventor 2023, San Francisco, CA, USA). The model consisted of a block (18.5 mm × 30 mm × 30 mm) and a layer simulating cortical bone with a thickness of 1 mm. Each 4-mm-diameter, 15-mm-long implant was virtually inserted into the 3D block at various angles (0°, 15°, and 20°). The shape of the implant body was then subtracted from the block to create the negative space corresponding to the implant. This process illustrates the optimal fit between the bone and the implant. Additionally, to apply the load to the implant, a cylindrical rod (2.82 mm × 10 mm) was created and inserted into the implant through the conical coupling according to Pammer et al.’s study [22]. Subsequently, the 3D model was imported in .stp format for analysis in the FEA software (ANSYS 2023 R1, Workbench, Canonsburg, PA, USA), as shown in Figure 1.

### 2.2. Materials

The physical and mechanical properties of bone are crucial factors in implant placement. The alveolar bone, acting as the site for implants, primarily consists of low-density cancellous tissue, along with a thin, dense cortical layer that absorbs most of the load. In contrast, the cancellous tissue provides vital support to prevent the cortical bone from collapsing. It is important to note that the mechanical properties of bone can change with age and other factors [39]. Bone tissue displays anisotropic characteristics, and the variation in Young’s modulus is linearly correlated with bulk density, as shown in Equations (1) and (2) [40].

(1) $$E_{cancellous}=1.904\times \rho^{1.64}$$

(2) $$E_{cortical}=2.065\times \rho^{3.09}$$

Furthermore, by utilizing computed tomography (CT), one can determine the distribution of Hounsfield units (HU) and relate it to density $\left(\rho \right),$ as shown in Equation (3) [40].

(3) $$\rho \left(\frac{g}{cm^{3}}\right)=0.0007918\;*\;HU+0.4718988$$

Extensive laboratory tests have been conducted to establish correlations between the experimentally determined density of bone samples (*ρ*) and mechanical properties such as Young’s modulus (E) and Poisson’s ratio (v), which serve as indicators of stiffness [41,42,43,44,45,46]. The relationships observed in these studies, resembling Equations (1) and (2), are summarized in Table 1 [22]. The titanium alloy Ti–6Al–4V, which has a Young’s modulus of 110 GPa and a Poisson’s ratio of 0.3, has been used as the material for the implants [24].

The classification system outlined in Table 1 offers an overview of the structures and mechanical properties of all bone classes, ranging from D1 to D4. Class D1, predominantly located in the anterior mandible, is characterized by its high density, mainly consisting of cortical bone tissue. In contrast, classes D3 and D4, found in the posterior mandible, are primarily composed of low-density trabecular tissue [34]. Generally, higher bone density correlates with increased strength and stiffness of the bone. However, this study only analyzed bone classes D2 and D3, in line with the in vitro analysis conducted by Comuzzi et al. [18]. Additionally, an isotropic approach—where the mechanical properties are uniform in all directions—may be utilized to simplify models and enhance simulations. This assumption is reasonable given the limited number of numerical studies that have characterized this type of tissue [47,48]. Thus, the materials simulated in this study were assumed to demonstrate linear elastic, homogeneous, and isotropic behavior.

### 2.3. Finite Element Analysis (FEA) Simulation

To conduct numerical simulations, implants were introduced into the blocks. To create the negative shape of the fixture, the dimensions of the implant were subtracted from the block. Finite element modeling was subsequently employed to represent the dense outer surface of the bone, known as cortical bone, using Solid 226 hexahedral elements. The use of hexahedral elements, defined by their 3D shape and six faces, allowed for accurate results while minimizing the number of elements needed. Additionally, Solid 187 tetrahedral elements were used to model the less dense trabecular bone. These tetrahedral elements are especially well-suited for representing irregular geometries and adapting to the complex architecture of trabecular bone [49,50,51,52,53]. A total of 163,424 elements and 23,497 nodes were used for each group.

**Figure 2:**
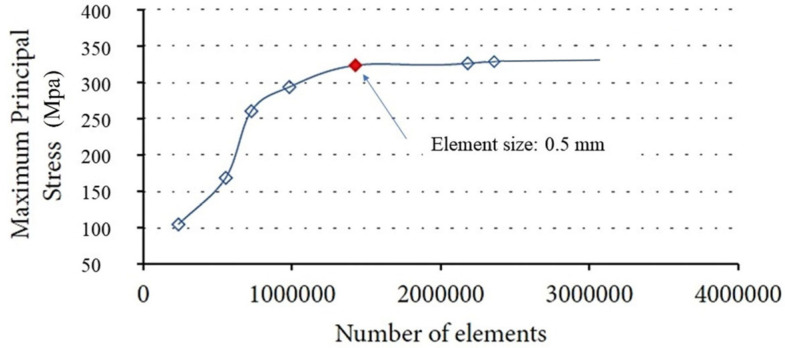
Results of the convergence analysis for the finite element model.

To assess the accuracy of the modeling, a mesh convergence analysis was conducted, illustrating the variation of stress based on the number of elements used (Figure 2).

**Figure 3:**
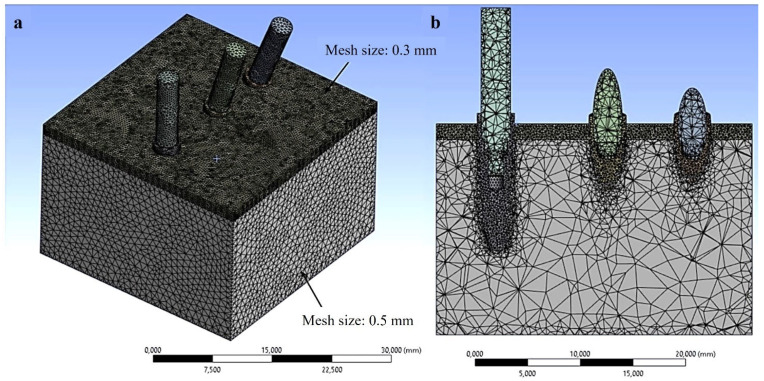
Discretization of the three-dimensional (3D) model of the inclined implant. (**a**) Complete model; (**b**) Cross-sectional view of the implants at the implant-bone contact.

The analysis shown in Figure 2 indicates that stress variation reached a plateau at the marked red point, corresponding to a mesh size of 0.5 mm. It can be concluded that the 0.5 mm element size demonstrated stability in predicting numerical results and provided an optimal balance between accuracy and computational efficiency. To enhance accuracy further, a refined mesh size of 0.3 mm was applied at the implant-bone interface and cortical layer, as illustrated in Figure 3. The finite element software ANSYS R1 2023 (Workbench, Canonsburg, PA, USA) and a computer equipped with a 13th-generation Intel Core i7 processor (Intel, Santa Clara, CA, USA) and 16 GB of RAM were used for this investigation.

### 2.4. Loads and Constraints

**Figure 4:**
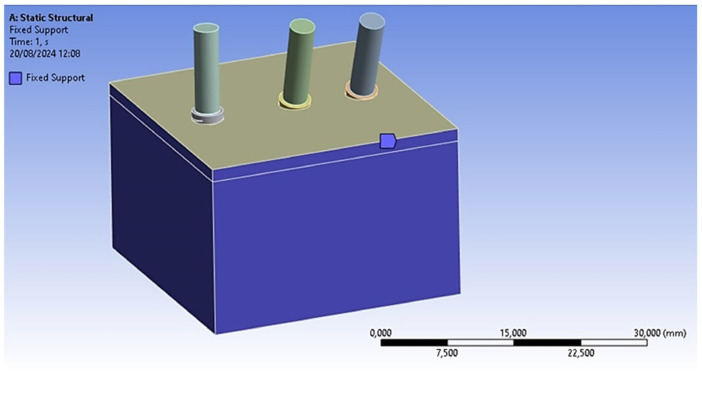
Constraint conditions of the block.

The subsequent phase involved defining the loading and constraint conditions within the finite element model to evaluate the mechanical response of the implant system under an applied force. As a result, all surfaces of the block, except for the top surface, were constrained in all directions. A frictional contact without separation was established between the implant and the block, characterized by a coefficient of friction of 0.3 [52]. Moreover, a fixed contact configuration was applied between the implant and the abutment (Figure 4).

**Figure 5:**
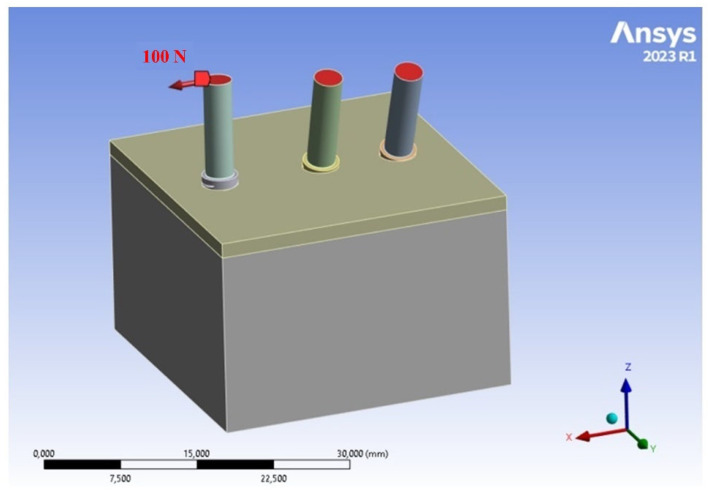
The 3D model of the implant under horizontal load application.

Figure 5 illustrates the application of a horizontal load of 100 N, placed on the top of the cylindrical supports of the implants. This load is not related to functional mastication. Instead, it enables a numerical assessment of ISQ and primary stability by calculating implant micro-mobility [22].

### 2.5. Numerical Method for Assessing the Implant Stability Quotient (ISQ)

To assess the ISQ parameter using the FEM, the following steps were taken:

- A horizontal load of 100 N was applied to inclined implants embedded in the bone;
- The micro-movements of the implant were evaluated using the FEM, concentrating on the micro-movements at the neck of the implants according to the direction of the applied load;
- Subsequently, the results of the micromovement analysis were incorporated into Equation (4) [22].

(4) $$ISQ=74.94-5.21*\ln\left(Micromovements-0.24\right)$$

Equation (4), which correlates ISQ and micro-movements (mm), is applicable within the elastic limits. The corresponding stress limits are 130 MPa for cortical bone and 13 MPa for trabecular bone, respectively [53,54]. Consequently, the von Mises criterion was used to evaluate the equivalent elastic strain, which is crucial in determining the valid application of Equation (4).

These results were compared to in vitro ISQ assessments using a No. 78 Smart Peg (Osstell AB, Gothenburg, Sweden). In the study conducted by Comuzzi et al. [18], a power analysis of the sample and an appropriate statistical analysis were performed to ensure the reliability of the measurements and the significance of the data.

## 3. Results

### 3.1. Analysis of Stress Fields in D3 Bone

**Figure 6:**
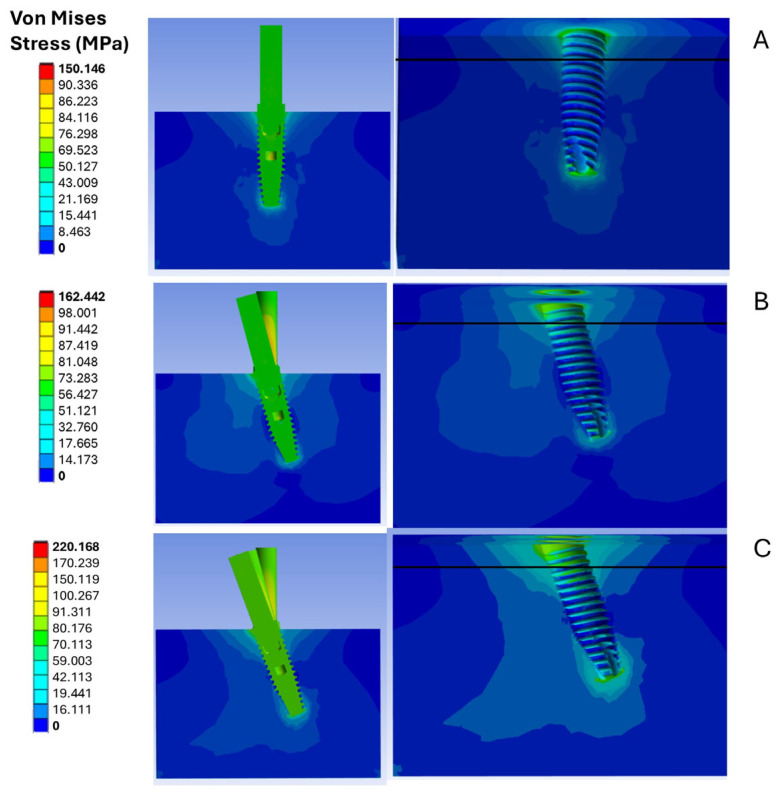
D3 bone stress analysis under three implant placement conditions: (**A**) straight implant (0°); (**B**) 15° inclined implant; (**C**) 20° inclined implant.

The analysis of the stress field developed in cortical bone is critical for determining whether externally applied loads on implants may pose a risk to the mechanical integrity of the bone. In this context, an evaluation was conducted to ascertain whether the stress levels achieved in the contact zone between the cortical bone and the implant remained below the plastic stress limit of the bone. As illustrated in Figure 6, the stress distribution between the implant and type D3 bone is presented.

This specific classification of bone has been selected for analysis due to extensive literature indicating that stress levels are often elevated in cases of low bone density [23,55,56,57]. It is crucial to note that cortical bone withstands greater stress than trabecular bone. Furthermore, observations indicate that the implant relieves a significant portion of the stress, a phenomenon commonly known as stress shielding.

It has been noted that stress levels increase with the inclination of the implant. Specifically, in model C, an implant inclination of 20° resulted in a peak stress of 220.2 MPa, compared to 150.1 MPa recorded for the straight implant model (A). Regarding cortical bone stress, model A showed a stress value of 55.4 MPa, while model B recorded 60.2 MPa. Finally, model C registered a cortical bone stress of 68.4 MPa. Based on the stress values obtained from the three different inclinations, it can be concluded that none of the conditions resulted in stresses significant enough to cause plastic deformation of the bone. Therefore, the loading conditions analyzed for the numerical evaluation of the ISQ values can be considered applicable.

### 3.2. ISQ Evaluation

**Figure 7:**
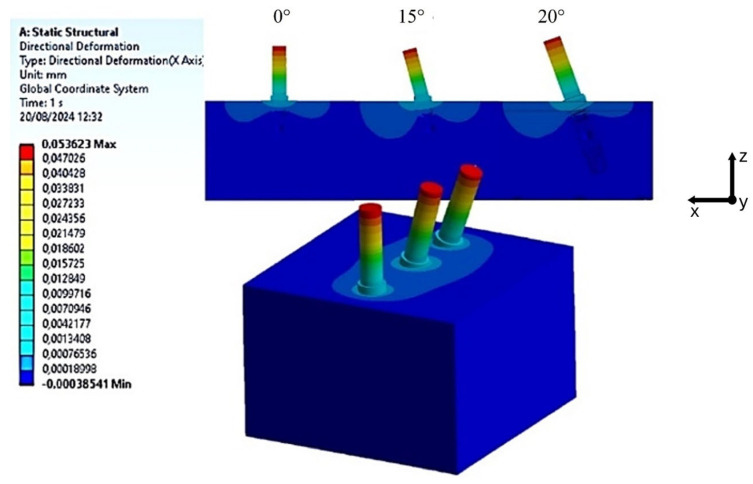
Distribution of horizontal displacements in the implants caused by a 100 N horizontal load applied to the D2-type bone block.

**Figure 8:**
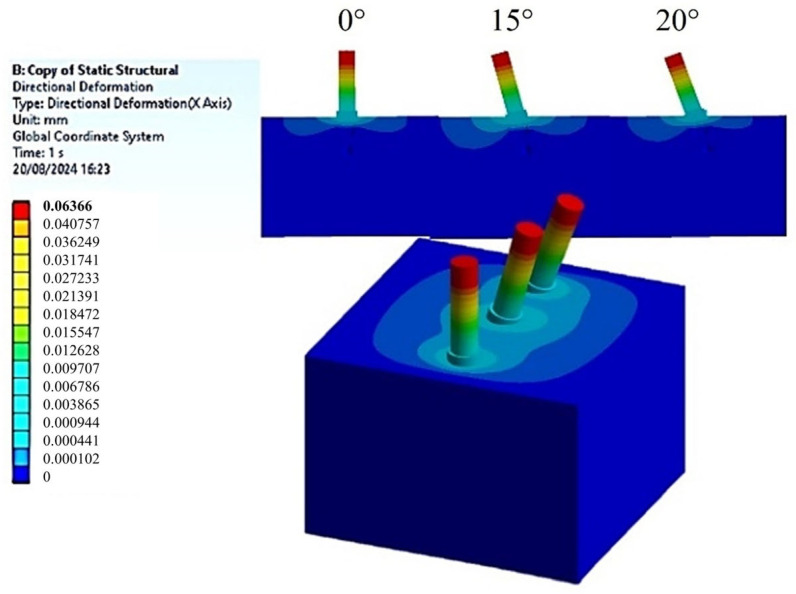
Distribution of horizontal displacements in the implants caused by a 100 N horizontal load for the D3-type bone block.

To assess the ISQ, the micro-displacements of the implant neck were measured along the *x*-axis, which indicates the direction of horizontal load application. These micro-displacements were analyzed for bone classifications D2 and D3, considering three different implant inclinations of 0°, 15°, and 20°, as shown in Figure 7 and Figure 8, respectively.

An initial examination of Figure 7 and Figure 8 indicated that the decrease in bone density from D2 to D3 correlates with an increase in micro-displacement of the implant along the x-direction. Specifically, for D2-type bone, a measurement of 0.05362 mm was recorded at the neck of the implant, compared to a maximum value of 0.06366 mm for D3-type bone. This observation aligns with established expectations, as a reduction in bone density is associated with diminished stability, compromising the mechanical integrity of the bone in supporting the implant [19,20]. By utilizing the relationship articulated by Pagliani et al. [58] in Equation (4) and based on the micro-displacement values derived from the FEA, micro-mobility was calculated. This methodology enabled the derivation of ISQ values, expressed as percentages, as detailed in Table 2.

Table 2 shows that the ISQ was considerably higher for D2-type bone compared to D3-type bone. Additionally, the ISQ increased with the inclination of the implants when exposed to a horizontal load. Notably, for D2-type bone, the ISQ of a vertically placed implant was recorded at 60.96, which rose to 61.10 when the implant was tilted by 20°.

To enable a comparison of the FEA-derived ISQ values, average ISQ values for bone classes D2 and D3 were compared with those obtained experimentally from an in vitro study conducted by Comuzzi et al. [59]. The results of this comparative analysis are presented in Table 3.

The results obtained from the two methodologies showed similar trends. Notably, the difference between the FEA results and the in vitro measurements was only 2.86% for D2-type bone and 1.27% for D3-type bone.

## 4. Discussion

The objective of this research was to compare data on implant stability obtained from a recent in vitro study using artificial bone blocks [59] with numerical simulations based on FEA. This comparison aimed to establish a correlation between the results derived from both in vitro and numerical bone models, thus validating the finite element simulation against in vitro experimental tests. Additionally, the study sought to evaluate the reliability of both methodologies while identifying their respective strengths and limitations. The primary focus of this investigation was to develop a more direct and accurate method for quantifying ISQ values through FEA and correlating these values with outcomes from in vitro biomechanical tests conducted on polyurethane, particularly concerning micro-mobility. Specifically, ISQ values of implants inserted at angles of 0°, 15°, and 20° were assessed based on mathematically calculated natural bone properties and systematically compared to in vitro results from polyurethane materials. The innovation of this study lies in applying FEM to assess the primary stability of dental implants, simulating micro-mobility under various conditions, and translating these simulations into measurable ISQ.

The ISQ can be assessed using radio frequency analysis, which employs a device to emit vibrations onto the implant and records its corresponding response. ISQ values serve as predictive indicators for determining the appropriate loading time for dental implants. Values exceeding 60 indicate medium to high stability, allowing for loading, whereas values below 60 suggest reduced stability, necessitating careful evaluation before loading the implant. Additionally, higher ISQ values correlate with a relatively stable implant during the critical healing phase, which is essential for successful osseointegration [43]. In a broader context, the stability of dental implants is influenced by a combination of anatomical, surgical, and prosthetic factors. A study conducted by Díaz-Sánchez et al. [60] demonstrated a positive correlation between bone quality and primary stability, with increased stability observed in male patients and in posterior regions of the jaw. Specifically, the classification of bone types showed a strong association with ISQ immediately following implant placement. The implant system used in this study featured a cylindrical design with a hydrophilic surface, which promotes early osseointegration. This combination may enhance implant stability and increase the likelihood of achieving long-term success in clinical settings. Additionally, the BIC serves as a critical parameter for evaluating the primary stability of dental implants, representing the direct contact between the bone and the implant surface, thus illustrating the integration of the implant with the surrounding bone. Various factors, including bone quality, implant shape, and dimensions, can influence the BIC [10,61].

Accurately emulating bone behavior is crucial for predicting implant stability. Currently, in vitro evaluations utilize polyurethane blocks and sheets to mimic bone structure [20,62,63]. This method is commonly used in research on the biomechanics and stability of dental implants, enabling a controlled assessment of implant performance. Additionally, polyurethane foams can be customized to replicate various bone densities [17,18,23]. For example, a study by Romasco et al. [17] demonstrated that the primary stability of implants is significantly affected by the shape, especially the length of the implant. This research also indicated that the density of polyurethane influences implant primary stability, with higher-density polyurethane blocks showing greater stability. Furthermore, other researchers have emphasized that conical implants offer improved stability compared to their cylindrical counterparts [64,65,66].

The direct method employed in in vitro testing with polyurethane to evaluate ISQ was developed for FEA based on an established correlation between primary stability and micro-movement. This relationship is only applicable within the elastic behavior range of the material, as discussed in the study by Pammer et al. [22]. This innovative methodology can be effectively utilized by both researchers and clinical practitioners involved in assessing implant performance using ISQ metrics.

The findings of this research indicated that the ISQ values obtained from experimental evaluations led by Comuzzi et al. [18] using polyurethane blocks were comparable to those derived from FEA simulations. Notably, the divergence between the FEA simulations and the in vitro data was less than 3%. Furthermore, it was observed that the ISQ values for purely horizontal loading increased with the inclination of the implant. Based on the FEA simulations conducted in this investigation, a significant finding relates to the dependence of implant stability on the characteristics of the surrounding bone structure, rather than being influenced by the type or inclination of the implant. For implants exhibiting low ISQ values, a thorough reassessment of their structural integrity is advisable. Conversely, implants that demonstrate high predicted ISQ values through FEA analysis may proceed to the prototyping phase.

From a mechanical perspective, the inclination of the implant increases stress in the surrounding bone [27,67,68]. For instance, a study by Nishimura et al. [69] found that a 15° inclination led to lower bone stress compared to a 45° angle, which saw a 45% increase in stress. Conversely, Guven et al. [70] revealed that inclined implants with short cantilevers could reduce stress on the cortical bone, thereby minimizing the generated moment of force and easing stress on adjacent bone tissues. A numerical analysis by Ibrahim et al. [71] showed that a 25° inclined implant caused greater deformation, measuring 13.286 μm in the bone, compared to a vertical implant. Additionally, research by Bevilacqua et al. [72] indicated that using distally angled implants with reduced or eliminated posterior cantilevers significantly decreased peri-implant stress, quantified as 12.9% for a 15° angle, 47.5% for a 30° angle, and 73.5% for a 45° angle.

This occurs because the bending of the implant intensifies as the inclination increases, resulting in greater stress on the bone. Specifically, the distribution of chewing forces changes significantly when an implant is placed obliquely compared to a vertically positioned implant. This alteration in force distribution can lead to an increase in compressive stresses within the trabecular bone. In particular, trabecular bone is better suited to withstand compressive loads due to its structural properties, which allow it to manage this type of force more effectively than tensile forces. Moreover, applying compressive forces is beneficial for stimulating osteogenesis—the process of new bone formation—and enhancing bone adhesion to the implant surface. This response is crucial for achieving and maintaining the primary stability of the implant, especially in scenarios where the surrounding bone density is low. Therefore, bone quality significantly impacts stress transmission. Generally, higher bone classes (D1 and D2) have greater density and can better support applied loads, distributing forces more evenly and lowering the risk of excessive stress, which subsequently results in reduced stress transmitted to the bone. In contrast, lower bone classes (D3 and D4) have lower density, potentially leading to increased forces concentrated around the implant and a higher risk of implant failure due to excessive stress. Few studies in the literature have compared inclination effects with bone quality. An in vitro study by Chun Wu et al. [73] noted that a greater inclination, coupled with a less favorable position (mesial supracrestal), contributed to diminished primary stability of the implants. However, this study also showed that placing the implant in a crestally favorable position resulted in a slight increase in implant stability as the inclination angle rose. Specifically, for a D2 bone class, a vertically placed implant recorded an ISQ of 60.96, while a 15° inclination yielded a slight increase to an ISQ of 61.10. When the angle exceeds 15°, a larger area of trabecular bone contacts the implant body, leading to enhanced support. Therefore, it can be concluded that there is no direct relationship between the inclination of the implant and its stability, as results can be affected by factors such as bone density and the implant’s position (supracrestal, crestal, or subcrestal).

It is essential to recognize that the precision of FEA modeling depends on several factors that influence its ability to produce reliable outcomes. These factors include the accurate representation of the analyzed structure (which encompasses geometry, materials, and boundary conditions), the precision of input data, and the quality of the mesh. An excessively coarse mesh may result in inaccurate results, while an excessively fine mesh can prolong computational time without yielding proportional improvements in accuracy. Thus, it is crucial to achieve a balance between computational efficiency and mesh precision through convergence analysis. Moreover, each FEA model necessarily incorporates assumptions and approximations that may affect the reliability of the results, including the mechanical characterization of materials. Consequently, the findings from this study require comprehensive validation, both in vivo and in vitro, due to several simplifying assumptions implemented during the analysis. Notably, while the bone was modeled as isotropic, its actual behavior is anisotropic, indicating that this simplification did not fully account for the complex and heterogeneous properties of human bone tissue [39]. Anisotropy suggests that the mechanical response of bone varies depending on the direction of the applied forces. Currently, there is no consensus on the appropriate values of mechanical properties to simulate various scenarios of bone density, which often leads to the assumption of isotropy for the sake of simplification and manageability in clinical situations with limited computational resources. However, studies have shown that anisotropy can increase the levels of stress and strain in cortical bone by 20–30% compared to isotropic models. For example, a study conducted by Liao et al. [74] demonstrated that the maximum value of stress and strain in a bone model exhibiting anisotropic behavior can increase by as much as 70% compared to an isotropic model. In another numerical study by Mahony et al. [75], it was reported that anisotropy caused a significant increase in stresses at the bone-implant interface, with normal stresses in the cortical region increasing by up to 20% in comparison to the isotropic model. Furthermore, this anisotropy led to a 3–4-fold increase in interface stress levels in cancellous bone compared to values obtained from the isotropic model, exceeding the permissible stress levels for the integrity of the bone structure. In a comparative study by Geraldes et al. [76], it was found that the isotropic model reached a stable solution in fewer iterations (26) compared to the orthotropic model (29 iterations). This suggests that while the isotropic model is less accurate in representing material properties, it converges to a solution more quickly. Regarding the distribution of stresses in various directions, the orthotropic model demonstrated superior capabilities compared to the isotropic model. While the literature provides data for the anisotropic characterization of cortical bone [77,78,79], information on trabecular bone remains limited. The only experimental study on trabecular bone in the human mandible proposed a model of transverse isotropy, with the axis of symmetry oriented in the inferior-superior direction [80,81]. Without a well-defined automated assignment procedure, generating the principal anisotropic orientations varying along the mandible poses a challenge. Consequently, many biomechanical studies tend to utilize simplified isotropic material laws, representing a limitation of this study that will be analyzed in the future alongside comparisons, including in vivo tests.

Furthermore, assuming a fixed contact between the implant and bone or polyurethane, which simulates perfect osseointegration, may introduce inaccuracies since osseointegration rarely reaches 100% effectiveness. Therefore, in future studies, considering a non-ideal interface that uses friction or non-linear contact models to better simulate the micromovement of the implant may be beneficial.

Moreover, employing polyurethane as a substitute for bone properties does not adequately replicate the biological and mechanical characteristics of human bone, particularly the viscoelastic response arising from hydration, which is lacking in polyurethane [82]. Consequently, the direct relevance of these findings to clinical practice may be limited, requiring further validation with actual bone tissue. Additionally, it is crucial to acknowledge the differences in test protocols, materials utilized, and environmental conditions between the two studies, as in vitro tests and numerical simulations inherently differ in their methodologies. Despite these variations, the ISQ results obtained from the numerical simulations showed a discrepancy of less than 3% compared to the ISQ data from the in vitro tests. This evidence indicates good reliability of numerical simulation and suggests that the simulations can effectively reflect the mechanical behavior of dental implants in clinical scenarios. However, statistical comparisons between the two results are not feasible because FEA results represent unique data linked to the chosen parameters intended for analysis; thus, it is not possible to reproduce the same results or expect different outcomes with the same set of parameters. To further strengthen the validity of the numerical model, future in vitro studies should employ more rigorous statistical analysis through multiple tests. This approach will enhance the model’s validation and contribute to greater confidence in the clinical applications of FEA simulations.

The clinical approach emphasizes techniques that enhance bone integrity and ensure long-term implant stability while avoiding extreme angles that may jeopardize implant health and function. Typically, angles between 15° and 30° are viewed as safer and more effective for maintaining implant stability and minimizing the risk of complications. An angle of 45° can complicate the insertion process and diminish controllability, potentially undermining the accuracy and initial stability of the implant. However, future studies may assess the ISQ implant stability index concerning implants positioned at steeper angles, such as zygomatic or pterygoid implants. Nevertheless, the discrepancies observed among the various configurations, all remaining under 3%, are deemed satisfactory for numerical analysis. Indeed, the literature indicates that acceptable error margins in finite element simulations across different software platforms typically remain below 15% for experimental data and below 6% for analytical data, demonstrating a high level of reliability [41,42,83,84,85,86,87,88]. The integration of FEA and in vitro studies analyzed in this research enabled a more comprehensive understanding of the mechanical interactions involved. While in vitro experiments validate specific conditions and parameters, FEA can extend these results to a broader range of clinical scenarios, offering insights that may not be achievable in a laboratory setting. Furthermore, the discrepancies observed between FEA predictions and in vitro results can serve as a vital feedback mechanism for refining the parameters and assumptions used in FEA simulations.

## 5. Conclusions

The primary finding of this study was that the ISQ of dental implants can be assessed through mechanical simulations, particularly focusing on micro-movements, using the FEM. The simulations revealed that implant stability is affected by several factors, including implant geometry, bone quality, and the specific angle of implant insertion. It is essential to note that the results obtained from the FEA derive from numerical simulations designed to replicate anticipated in vivo behavior. Furthermore, the study demonstrated that in vitro testing with polyurethane and FEA yielded comparable results. This analysis suggested that the FEM may be beneficial for evaluating new dental implant designs during their developmental phases, providing a quicker and more cost-effective way to predict quantified ISQ across different bone qualities compared to polyurethane in vitro testing. However, the two methods can be seen as complementary for a deeper understanding of the biomechanical behavior of dental implants.

Future research should focus on conducting a prospective study that concurrently assesses the three models—namely, in vitro, numerical, and in vivo—to improve confidence in the accuracy of outcome predictions across both methodologies.

## Figures and Tables

**Table 1:** Mechanical properties of bone.

Mechanical Properties	Cortical Bone	D1 Bone	D2 Bone	D3 Bone	D4 Bone
Density (ρ, kg/m^3^)	1600	800	640	480	320
Young’s Modulus (E, MPa)	16,000	9500	5500	1600	690
Poisson’s Ratio (v)	0.3	0.3	0.3	0.3	0.3

**Table 2:** Implant stability quotient (ISQ) values determined using Equation (4) with the finite element analysis (FEA) results.

Material	ISQ (0°)	ISQ (15°)	ISQ (20°)
D2-type bone	60.96	61.05	61.10
D3-type bone	55.68	55.77	55.90

**Table 3:** Comparison of the average ISQ determined through FEA with in vitro testing results.

Material	Average ISQ Through FEA	Average ISQ Through *In Vitro* Testing [59]
D2-type bone	61.10	62.90
D3-type bone	55.78	56.50

## Data Availability

All experimental data to support the findings of this study are available in the manuscript or upon request by contacting the corresponding author.
